# Once upon a time there was complex numerical estimation

**DOI:** 10.3389/fnhum.2012.00300

**Published:** 2012-11-05

**Authors:** Christian Agrillo

**Affiliations:** Department of General Psychology, University of PadovaPadova, Italy

During the last decade, evidence collected in cognitive, developmental, and comparative research showed that adults prevented from verbal counting, along with infants and non-human animals possess numerical systems that are independent of language (the so-called “non-verbal numerical abilities”). Interestingly very similar results were reported among mammals, birds, and fish, leading some authors to believe in the existence of the same numerical systems shared among vertebrates (Feigenson et al., 2004; Beran, 2008; Agrillo et al., 2012). However, the exact nature of these capacities is unknown and it is currently unclear whether or not the similar performance described in the literature is the result of a common origin of cognitive skills, or instead reflects independent convergent evolutions.

Some of these comparative studies have recently received a lot of media coverage, as they suggested that numerical discrimination is not only a vertebrates' prerogative. Bees, for instance, proved able to reach a food reward, apparently by enumerating the landmarks encountered sequentially during flight (Dacke and Srinivasan, 2008); bees can also make use of numbers in a sequential matching-to-sample task (Gross et al., 2009). Ants reportedly pass numerical information to other ants when transferring information about which branch of a maze contains food (Reznikova and Ryabko, 2011) and spiders (Nelson and Jackson, 2012) are believed to base their settling decisions in nest selection on the number of already settled conspecifics, preferring to join nests where only one conspecific is present (instead of 0, 2, or 3 conspecifics).

The idea that organisms with such a small brain size can somehow process numerical information has opened a wide debate in the scientific community as to whether these studies have properly controlled for non-numerical continuous variables that co-vary with numbers (i.e., cumulative surface area, density, overall space occupied by the groups, etc.). After all, the potential implications of these works could not be underestimated by neuroscientists: the very idea that higher-level cortical mechanisms are a sine qua non condition for number processing is at risk. The study by Stoianov and Zorzi (2012), which is based on computational models, has now provided a potential explanation for the astonishing numerical abilities of invertebrates. The authors used deep networks; that is, a multilayer neural system that shares top-down and bottom-up connections to infer perceptions of the sensory input. The study investigated sensitivity to numerical information in terms of internal coding by hidden neurons after learning (the network had one visible layer encoding sensory data and two hidden layers hierarchically organized). The results showed that highest-level populations of as few as 35 hidden neurons were able to support the process of numerosity estimation. This implies that numerosity might be potentially extracted with the use of a very limited number of neurons, definitively far fewer neurons than was previously thought (Dehaene and Changeux, 1993). It is important to note that the response of hidden neurons was not initially stipulated; just the opposite, it represented an unsupervised emergent property. Stimuli—patterns representing objects differing in numerosity—were strictly controlled for non-numerical continuous quantities that co-vary with numbers (such as cumulative surface area, shape, size, and density of the objects), thus preventing the possibility that results were due to a more general ability to estimate continuous amounts. Even more remarkably, the model's deepest layer proved to be able to support human-like performance, as the internal Weber fraction in relative numerosity judgments strictly resembled that commonly observed in humans.

The idea that numerosity estimation can spontaneously emerge as a statistical property aligns with a previous study on adult humans which suggests that the ability to estimate the number of items might be a sort of primary visual property of stimuli, such as color and contrast, that is based on a low-level mechanism (Burr and Ross, 2008). Above all, the conclusions of Stoianov and Zorzi (2012) are in line with a recent theoretical view that emphasizes the importance of neural circuits, rather than the size of brain regions that are supposed to modulate higher cognitive functions (Chittka and Niven, 2009).

These results may also have implications in the theoretical debate that surrounds non-verbal estimation of other magnitudes, such as time and space. According to Walsh (2003), time, space, and number would be processed by a common magnitude system (a theory of magnitude which is commonly called “ATOM”) that is mainly located in the parietal lobe. The theory has been primarily investigated by using either contrast paradigms (Agrillo et al., 2010) or the observation of neuro-anatomical correlates (Cohen Kadosh et al., 2008). Computational models could provide useful insights as well. Indeed, one potential prediction of ATOM would be that, just as emergent numerosity detectors are now recognized, emergent space, and time detectors should be reported. In addition, if there is a common system, then the same number of neurons should be required to process the three magnitudes. Any significant difference would suggest at least a partial independence in processing these magnitudes.

Of course, the model created by Stoianov and Zorzi (2012) also needs to be tested in a wider range of contexts. In their research, stimuli consisted of 30 × 30 pixel images that contained from 1 to 32 rectangular figures. None of the figures overlapped each other, and they were all separated by at least one pixel. However, in everyday life—for instance, when we have to select a queue that contains the least amount of people—stimuli can move incessantly in a tridimensional space, modifying their inter-individual distance or changing orientation and, hence, the visible area. They can also partially to totally occlude each other. The numerosity estimation of natural stimuli might be processed by a larger neural network than that suggested by the authors. In line with this argumentation, neuroimaging studies support the idea that humans recruit distinct brain areas that involve, but are not limited to, inferior parietal regions to estimate numerosity (for meta-analysis, see Arsalidou and Taylor, 2011). It is worth noting that the model presented only a 30 × 30 pixel resolution, and that numerosity detection is primarily based on a signal from center-surround neurons that are described in the early visual system. The possibility remains that the quantity of numerosity detectors might increase while increasing the visual resolution of the model. From a theoretical point of view, it is possible that organisms that have a small brain size and reduced visual resolution might be properly equipped with the few numerosity detectors which were described in Stoianov and Zorzi's study (2012), while humans and other species that have better visual resolution might display more numerosity detectors. Future studies are required in order to test this hypothesis. In this sense, the model that is described in their paper might be considered a very useful formal model of human abstract numerosity estimation that captures key elements—namely, center-surround filtering and normalization with global visual properties of human numerosity estimation. However, some details need to be further clarified with respect to the visual processes that are involved in numerosity estimation.

One thing is certain: the study by Stoianov and Zorzi (2012) now paves the way for further investigation into the computational bases of numerosity estimation within non-human animals. Specific deep networks might be set up according to the peculiarities of different species (i.e., that take into account the different number of sensory and internal neurons), helping us to shed light on the true nature of the similar performance reported in the literature: similar results with respect to the computational bases would support the “common origin” hypothesis of non-verbal numerical abilities, while the opposite pattern of data would support rather the “convergent evolution” hypothesis.

We can only speculate about this latter point, but at least the lesson we can currently draw is clear: 35 units can spontaneously learn how to extract numerical information. This is enough even for simpler organisms than those reported in the literature. The last decade of comparative psychology was characterized by the discovery of numerical abilities in almost all vertebrate species. In light of the conclusions of Stoianov and Zorzi (2012), we must now expect the coming decade to be epitomized by a wide-scale invasion of invertebrates into the numerical cognition literature.
